# Daytime Sleepiness and Quality of Sleep in Patients with COPD Compared to Control Group

**DOI:** 10.5539/gjhs.v5n3p150

**Published:** 2013-02-25

**Authors:** Mohammad Ali Zohal, Zohreh Yazdi, Amir Mohammad Kazemifar

**Affiliations:** 1 Pulmonologist, Assistance Professor of Qazvin University of Medical Sciences, Qazvin, Iran; 2 Occupational Medicine Specialist, Metabolic Disease Research Center, Assistance Professor of Qazvin University of Medical Sciences, Qazvin, Iran; 3 Clinical Toxicologist, Assistance Professor of Qazvin University of Medical Sciences, Qazvin, Iran

**Keywords:** COPD, quality of sleep, Pittsburgh questionnaire, day time sleepiness

## Abstract

**Objectives:**

Chronic obstructive pulmonary disease (COPD) is a widespread disease. It produces some night symptoms such as nighttime cough and dyspnea. Then subjective and objective changes in sleep pattern are expected. Present study was conducted to determine frequency of sleepiness and quality of sleep in patients with COPD.

**Materials & Methods:**

Present case-control study has been performed on 120 patients with diagnosis of COPD who had been referred to pulmonary disease clinic in a University teaching hospital. One hundred twenty age- and sex- matched healthy individuals were recruited in the study and served as control. Spirometry (PFT) was performed for all patients. Patients were categorized under 3 groups in relation to their PFT as follow: mild COPD (FEV1/FVC<70% and FEV1≥80%), moderate COPD (FEV1/FVC<70% and 50%≤FEV1<80%), and severe COPD (FEV1/FVC<70% and FEV1<50%). Pittsburgh Sleep Quality questionnaire (PSQI) and Epworth Sleepiness Scale (ESS) were used to estimate quality of sleep and daytime sleepiness in the patients and control group. The collected data were analyzed using version 16 SPSS software. Student’s T- test, Chi- square and multiple logistic regressions were used as appropriated.

**Results:**

120 patients with COPD (79 males and 41 females) and 120 normal individuals responded to the questionnaires. Mean scores of quality of sleep were 8.03±3.66 and 4.2±2.8 in COPD patients and control group respectively. 32.1% of the patients had good sleep quality (PSQI score less than 5) and 67.9% had poor sleep quality. Daytime sleepiness (ESS≥ 10) was present in 34.8% of the patients and 15% of control people. Multiple logistic regressions showed that the patients reported significantly worse sleep quality and more daytime sleepiness than control group [OR=2.9; 95% CI (1.6-3.7) & OR=3.5; 95% CI (2.5-4.3) respectively].

**Conclusion:**

Results of present study confirmed that COPD is associated with daytime sleepiness and poor quality of sleep, possibly attributable to nighttime respiratory difficulties and concomitant sleep apnea. Assessment of the patients for symptoms of sleep apnea, daytime sleepiness should be a part of regular follow up visits of patients with COPD.

## 1. Introduction

Chronic obstructive pulmonary disease (COPD) is a widespread disease particularly with increase in age. It is estimated that 8-10 percent of adult population suffers from COPD (Owens et al., 2010; Løkke et al., 2006; Brutsche et al., 2006; Sanders et al., 2003). COPD exerts some impacts on quality of life of the patients as an unrelieved incapacitating disease (Vlipour et al., 2011).

The disease produces some night symptoms such as nighttime cough and dyspnea. Then subjective and objective changes in sleep pattern are expected. Nocturnal blood oxygen saturation decreases as a result of COPD, to end with pulmonary hypertension, cardiac arrhythmia, and patient’s arousal (Pauwels et al., 2001; Fleatham, 2003).

There are few studies about sleep disorders in COPD patients (Chaouat et al., 1995; Klink et al., 1987 & 1994). They have implied that patients’ symptoms may influence quality of sleep in patients with COPD (Chervin et al., 2000; Scharf et al., 2010). Study on patients with COPD has demonstrated that 70% of the patients have had poor quality of sleep (PSQI>5) (Nunes et al., 2009). In another study conducted to evaluate quality of sleep in 100 patients with COPD compared to control group, patients with COPD had shorter sleep duration, more delay in start of sleep, more frequent night awakening and lower nighttime oxygen saturation (Lofdahl et al., 2008).

There is no study about the matter among Iranian population. Present study was conducted to determine frequency of sleepiness and quality of sleep in patients with COPD and compare them with control.

## 2. Methods

Present case-control study has been performed on patients with diagnosis of COPD who referred consecutively to pulmonary disease clinic in a University teaching hospital, Qazvin city, in central part of Iran during Mar 2012 to Apr 2012. In this study, inclusion criteria in patients group were: 1) age more than 40 years; 2) Pulmonologist-diagnosed COPD according to history of smoking, airflow obstruction on spirometry, and the presence of at least one symptom related to COPD; 3) no hospitalization and no exacerbation within one month prior to the study. Exclusion criteria in both patients and control groups were: 1) history of other major pulmonary diseases; or 2) history of another chronic disease.

The patients were initially evaluated by a pulmonologist for assessment of severity of their disease. Their pulmonary function test (PFT) was assessed according to American thoracic medicine society. They were categorized under 3 groups in relation to their PFT results as follow: mild COPD (FEV1/FVC<70% and FEV1≥80%), moderate COPD (FEV1/FVC<70% and 50%≤FEV1<80%), and severe COPD (FEV1/FVC<70% and FEV1<50%). The patients provided informed consent for participation into the study.

120 patients were included in the study. Another 120 individuals with comparable age and gender were also selected and served as control. Demographic data of the patients include their age, gender, level of education, history of cigarette smoking, and night snoring were inquired and recorded.

Pittsburgh Sleep Quality questionnaire (PSQI) was used to estimate quality of sleep in the patients. The questionnaire has 89.6% sensitivity and 86.5% specificity. Patient’s opinion about his/her sleep quality during last 4 weeks was asked in this questionnaire. Finally 0-3 score was given in 7 sections according to Likert scoring; so total score of the patients would be 0-21. Score more than 5 means poor sleep quality (Buysse et al., 1989).

Epworth Sleepiness Score (ESS) was employed to evaluate daytime sleepiness in the participants. The questionnaire comprises 8 questions which define situations that in them the patient may face with sleepiness inadvertently. Each question takes 0-3 score. If total score of the patients is more than 10, he/she experience more than standard daytime sleepiness (Dohns, 1999).

The collected data were analyzed using SPSS software version 16. Quantitative parameters were reported as mean± SD. Qualitative parameters were reported as number and percent. T-test, chi-square test and multiple logistic regressions were used to evaluate relationship between parameters.

## 3. Results

120 patients with COPD (79 males and 41 females) and 120 normal individuals responded to the questionnaires. Mean age of the patients was 66.4±10.8 years. Their mean BMI was 25.91±4.97.

Demographic characteristics of the patients group and control group have been demonstrated in Table 1. Summary of notable demographic variables of the patients and results of analysis of the filled questionnaires sorted by severity of their disease have been displayed in Table 2.

**Table 1:** Demographic characteristics of the patients group and control group

Variables	Patients with COPD (n=120)	Control group (n=120)	P-value
age	10.8±66.4	6.4±57.3	0.21
Sex:			
male	79 (65.8%)	65 (54.1%)	0.09
Female	41 (34.2%)	55 (45.8%)	
Marital status:			
single	29 (24.2%)	18 (15%)	0.2
married	91 (75.8%)	102 (85%)	
Level of education (years)	3.7±7.6	4.1±9.2	0.07
BMI	4.9 ± 25.9	3.2 ±27.1	0.08
Cigarette smoking:			
yes	61 (50.8%)	37 (30.8%)	0.06
no	59 (49.2%)	83 (69.2%)	
Daytime sleepiness	38 (31.6%)	18 (15%)	0.01
Night snoring	58 (48.3%)	23 (19.1%)	0.002

**Table 2:** Notable demographic variables of the patients and results of analysis of the filled questionnaires sorted by severity of their disease

Variables	Mild COPD (n=20)	Moderate COPD (n=58)	Severe COPD (n=42)	P-value
age	57.8±12.1	6.9±62.7	10.3±69.4	0.07
Sex:				
male	8 (6.6%)	41 (34.1%)	30 (25%)	0.12
Female	12 (10%)	17 (14.1%)	12 (10%)	
BMI	3.7±25.8	4.1±26.1	5.8±25.2	0.08
FEV1	0.5±1.76	0.9±1.42	0.4±0.91	0.02
Daytime sleepiness	3.2±9.9	4.7±9.1	4.1±12.1	0.04
Night snoring:				
yes	18 (15%)	25 (20.8%)	15 (12.5%)	0.07
no	2 (1.6%)	33 (27.5%)	27 (22.5%)	
Sleep quality	5.3±7.8	6.1±8.3	3.4±9.1	0.03
Subjective sleep quality	0.6±1.3	0.4±1.6	0.6±1.7	0.06
Delay in initiation of sleep	0.7±1.0	0.5±1.1	0.6±0.9	0.1
Sleep duration	0.6±1.1	0.7±0.9	0.8±1.3	0.08
Sleep satisfaction	0.8±1.2	0.5±1.4	0.9±1.5	0.04
Sleep disturbance	0.5±1.7	0.3±1.4	1.1±2.1	0.02
Use of hypnotic drugs	0.7±1.8	0.4±1.7	0.7±2.2	0.05
Disturbance in daytime activities	0.9±1.8	0.5±1.7	0.9±1.9	0.09

67.9% of the patients had poor sleep quality as said by their Pittsburgh questionnaire, whereas only 27.5% in control group had poor sleep quality. Mean scores of quality of sleep were 8.03±3.66 and 4.2±2.8 in COPD patients and control group respectively. Detailed results of analysis of Pittsburgh questionnaire have been shown in Table 3. Results from multiple logistic regressions showed that patients have reported significantly worse sleep quality than control group [OR=2.9; 95% CI (1.6-3.7)].

**Table 3:** Results of analysis of Pittsburgh questionnaire in studied patients

	Patients group	Control group	OR (95%CI)	P-value
Total score of PSQI	8.03±3.66	4.2±2.8	2.9 (1.6-3.7)	0.02
Subjective sleep quality	1.9±0.8	1.4±0.7	1.9 (1.1-2.6)	0.03
Delay in initiation of sleep	1.3±0.5	0.9±0.7	2.1 (1.3-2.9)	0.04
Sleep duration	1.5±0.6	1.3±0.8	1.5 (0.6-2.1)	0.2
Sleep satisfaction	1.4±0.9	0.9±0.4	3.1 (1.9-4.2)	0.009
Sleep disturbance	2.1±1.1	1.9±0.6	1.6 (0.8-1.9)	0.07
Use of hypnotic drugs	2.2±0.7	1.2±0.5	2.6 (1.5-3.7)	0.007
Disturbance in daytime activities	1.9±0.9	1.1±0.3	2.5 (1.3-3.6)	0.005

Mean total ESS score was 9.9±3.4 and 6.8±2.6 in COPD patients and control group, respectively. Also daytime sleepiness was significantly higher in COPD patients compared to control people [OR=3.5; 95% CI (2.5-4.3)].

## 4. Discussion

Previous studies showed that the quantity and quality of sleep influences quality of life in chronic diseases such as COPD (Hynninen et al., 2007; Cully et al., 2006). It has been claimed that sleep disorders is the third determinant of quality of life in patients with COPD, after dyspnea and tiredness (Cavalcante et al., 2012). Consequently, presence of sleep disorders and evaluation of sleep quality should be noticed in patients with COPD.

Present study evaluated quality of sleep in patients with COPD using Pittsburgh questionnaire (PSQI). Mean scores of quality of sleep were 8.03±3.66 and 4.2±2.8 in COPD patients and control group respectively. 32.1% of the patients had good sleep quality (PSQI score less than 5) and 67.9% had poor sleep quality. The corresponding figures were 72.5% and 27.5% for control group respectively. PSQI score has been 11±5.4 in a group of COPD evaluated by Scharf; which denotes poor sleep quality in them (Scharf et al., 2010). Another study has confirmed that COPD patients had more delay in start of sleep, more frequent night awakening and lower nighttime blood oxygen saturation, compared to control group (Lofdahl et al., 2008). Present study again corroborated that poor sleep quality is quite prevalent in COPD patients.

COPD patients with more severe disease had poorer sleep quality in current study, since lower FEV1 went along with higher PSQI score. It is consistent with a study that has illustrated more severe diurnal hypoxemia is correlated with lower sleep effectiveness score in polysomnography (McSharry et al., 2012).

Decline in sleep quality is also seen in other conditions that are concomitant with nighttime hypoxemia such as asthma and emphysema. Sleep is adjusted by various neurotransmitters such as nor-adrenaline, melatonin, acetyl choline, serotonin, histamine, and dopamine. Hypoxemia may affect sleep characteristics through changes in the level of these neurotransmitters. Several animal and human studies have suggested that nighttime oxygen therapy recovers sleep quality in the patients (Glodstein et al., 1984; Ray et al., 2011).

COPD patients had lower PSQI score and sub-scores in present study; compared to control. Their sleepiness was higher too. Similar studies have reached to the same conclusion too. Studies conducted on patients with COPD and chronic bronchitis have reported more frequent night snoring and daytime sleepiness in the patients matched up to control (Koutsourelakis et al., 2008; Karachaliou et al., 2007). Sleep fractioning, concomitant sleep apnea and prescribed drugs may aggravate the problem (Nunes et al., 2009; Lofdahl et al., 2008; Buysse et al., 1989). Prevalence of COPD has been higher in patients with diagnosis of sleep apnea in a sleep clinic. Association between two diseases has estimated 11-16% (Chaouat et al., 1995; Resta et al., 2002). The term “Overlap Syndrome” was used to describe the combination of chronic obstructive pulmonary disease and obstructive sleep apnea syndrome (Weitzenblum et al., 2008).

Limitations of this study need to be mentioned. It seems more convenient that polysomnography would be used for evaluation of sleep in the patients, however technical limitations preclude this. Also determination of disease severity was performed by PFT. Assessment of arterial CO_2_ concentration seems more appropriate. Again it is an invasive procedure and was discarded.

## 5. Conclusion

In summary, results of present study confirmed that COPD is associated with poor quality of sleep, possibly attributable to nighttime respiratory difficulties and concomitant sleep apnea. Assessment of the patients for symptoms of sleep apnea, daytime sleepiness and other sleep disorders should be taken into account during regular follow up visits of patients with COPD.
